# Susceptibility to childhood onset rheumatoid arthritis: investigation of a weighted genetic risk score that integrates cumulative effects of five genetic risk variants

**DOI:** 10.1186/1546-0096-10-S1-A116

**Published:** 2012-07-13

**Authors:** Sampath Prahalad, Milton Brown, Susan D Thompson, Michael Zwick, David Cutler, Lori A Ponder, Jennifer Prozonic, Sheila Angeles-Han, Larry B Vogler, Christine W Kennedy, Carol A Wallace, Carol Wise, Marilynn G Punaro, Ann M Reed, Jane L Park, Elizabeth D Mellins, John F Bohnsack, David N Glass

**Affiliations:** 1 Childrens Hospital Medical Center, Cincinnati, OH, USA; 2 Emory Children's Center, Decatur, GA, USA; 3 Emory Children's Center, Atlanta, GA, USA; 4 Emory University, Atlanta, GA, USA; 5 Emory University School of Medicine, Atlanta, GA, USA; 6 Mayo Clinic, Rochester, MN, USA; 7 Seattle Children's Hospital & Regional Medicine, Seattle, WA, USA; 8 Stanford University Medical Center, Stanford, CA, USA; 9 Stanford University Medical Center, San Jose, CA, USA; 10 Texas Scottish Rite Hospital, Dallas, TX, USA; 11 Texas Scottish Rite Hospital for Children, Dallas, TX, USA; 12 University of Utah, Salt Lake City, UT, USA

## Purpose

Rheumatoid arthritis (RA) is an inflammatory arthritis with a peak onset age of ~50. Children with rheumatoid factor and/or anti-citrullinated peptide antibody positive juvenile idiopathic arthritis resemble adults with RA, and represent the childhood onset of RA (CORA). Variants of the genes encoding human leukocyte antigens (HLA) and several other genes are associated with RA, but have not been evaluated in children. To test the hypothesis that RA associated variants would also be associated with CORA, we investigated these variants in the largest cohort of children with RA. In order to understand the role of aggregate genetic risk factors, rather than associations of individual alleles with CORA, we investigated cumulative effect of 6 variants at 5 validated RA loci for association with CORA.

## Methods

155 Non-Hispanic White children with CORA and 410 ethnicity matched autoimmunity-free adult controls were typed for 5 confirmed RA-associated single nucleotide polymorphisms (SNPs) at four loci: rs6679677 in *PTPN22*, rs3761847 in *TRAF1/C5*, rs7574865 in *STAT4*, rs6920220 and rs10499194 in *TNFAIP3* loci. In addition, high resolution HLA-DRB1 genotypes were determined on 131 White cases and 100 White controls. We performed case-control tests to investigate the loci individually for allelic and genotypic associations using PLINK. We also calculated a weighted genetic risk score (wGRS) using these loci, with weights based on the natural log of the genotypic odds ratios (OR) for HLA-DRB1 alleles, and of published allelic OR for non HLA risk alleles, to compare cases and controls. Subjects were divided into seven categories of wGRS, that were +/- 0.25, 0.75 and 1.25, and >1.25 SD from the mean wGRS among controls.

## Results

The average onset age of CORA was 11 years, and 86% of cases were females. As anticipated there was a highly significant association between CORA and HLA-DRB1 alleles associated with RA. Allelic association tests revealed a significant association between CORA and *TNFAIP3* SNP rs10499194 [allelic OR 0.58 (0.42-0.81), p <0.001]. In addition we also found an association between CORA and the *PTPN22* [OR 1.48 (95%CI 1.01-2.19), p <0.05], and a marginal association with the *STAT4* [OR 1.32 (0.98-1.76), p <0.06] variants. The wGRS based on *HLA-DBR1* and the 5 RA-associated SNPs was significantly different between cases and controls (P < 5 X 10^-10^ by two sample T-test). When we excluded the *HLA* and examined the wGRS using only the 5 RA associated SNPs, the two groups were still significantly different (p < 7 X 10^-5^). Cases with wGRS >1.25 SD of the mean had a significantly higher OR of CORA (OR = 5.1 (1.9-13.4) referent to the population average. When we compared individuals in the extreme categories of wGRS, those in group 7 (>+1.25 SD from mean) had an OR of 6.8 (1.3-35.1) compared to those in group 1 (>-1.25 SD from mean).

## Conclusion

We have demonstrated that in addition to HLA-DRB, RA associated SNPs in *TNFAIP3*, *STAT4* and *PTPN22* are also associated with childhood onset RA. The magnitude and direction of association is similar between RA and CORA. Utilizing a combination of 6 risk alleles into a single wGRS, we have demonstrated the cumulative effect of HLA variants and RA associated variants in the susceptibility to childhood onset RA.

## Disclosure

Sampath Prahalad: None; Milton Brown: None; Susan D. Thompson: None; Michael Zwick: None; David Cutler: None; Lori A. Ponder: None; Jennifer Prozonic: None; Sheila Angeles-Han: None; Larry B. Vogler: None; Christine W. Kennedy: None; Carol A. Wallace: None; Carol Wise: None; Marilynn G. Punaro: None; Ann M. Reed: None; Jane L. Park: None; Elizabeth D. Mellins: None; John F. Bohnsack: None; David N. Glass: None.

